# The effects of measurement parameters on the cancerous cell nucleus characterisation by atomic force microscopy in vitro

**DOI:** 10.1111/jmi.13104

**Published:** 2022-04-21

**Authors:** Jiajing Zhu, Yanling Tian, Jin Yan, Jing Hu, Zuobin Wang, Xianping Liu

**Affiliations:** ^1^ School of Engineering University of Warwick Coventry UK; ^2^ Ministry of Education Key Laboratory for Cross‐Scale Micro and Nano Manufacturing Changchun University of Science and Technology Changchun China; ^3^ International Research Centre for Nano Handling and Manufacturing of China Changchun University of Science and Technology Changchun China

**Keywords:** A549 cells, AFM, mechanical properties, morphology, Young's moduli

## Abstract

Cancer is now responsible for the major leading cause of death worldwide. It is noteworthy that lung cancer has been recognised as the highest incidence (11.6%) and mortality (18.4%) for combined sexes among a variety of cancer diseases. Therefore, it is of great value to investigate the mechanical properties of lung cancerous cells for early diagnosis. This paper focus on the influence of measurement parameters on the measured central Young's moduli of single live A549 cell in vitro based on the force spectroscopy mode of atomic force microscopy (AFM). The effects of the measurement parameters on the measured central Young's moduli were analysed by fitting the force–depth curves utilising the Sneddon model. The results revealed that the Young's moduli of A549 cells increased with the larger indentation force, higher indentation speed, less retraction time, deeper Z length and lower purity percentage of serum. The Young's moduli of cells increased first and then decreased with the increasing dwell time. Hence, this research may have potential significance to provide reference for the standardised detection of a single cancerous cell in vitro using AFM methodologies.

## Lay description

Cancer is now responsible for the majority leading cause of death worldwide and it is noteworthy that lung cancer has been recognised as the highest incidence (11.6%) and mortality (18.4%) for combined sexes among a variety of cancer diseases. Therefore, it is of great value to investigate the mechanical properties of lung cancerous cells for early diagnosis. This paper primarily investigated the morphological properties and the influence of measurement parameters on the measured local elastic moduli of single live A549 cell in vitro using the AFM‐based force spectroscopy mode. In practice, there are many factors for incorrect or inaccurate experimental results using AFM to measure the characteristics of live cells, such as non‐homogeneous nature of cells, probe geometry and size, mechanical analysis model, substrate stiffness and different measurement parameters. The various measurement parameters have become the huge impact factor to influence the measurement result. Hence, this research may have potential significance to provide reference for the standardised detection of a single cancerous cell in vitro using AFM methodologies.

## 1 INTRODUCTION

Cancer is now responsible for the major leading cause of death worldwide. It is noteworthy that lung cancer has been recognised as the highest incidence (11.6%) and mortality (18.4%) for combined sexes among a variety of cancer diseases.^1^ Macroscopically, the reasons for cancer generation primarily ascribe to the socioeconomic development in regional variations, such as the aging of the population, emerging economies and lifestyle changes.

Cellular mechanics is the basis of cell and tissue engineering, which covers cell morphology, migration, differentiation, proliferation, adhesion and so on.^2^ In order to regulate the characteristics of cell morphology, proliferation and other functions, the cellular biological and mechanical properties with multiple stimulation are measured based on the physical and chemical principles of sound, light, heat, electricity, magnetism and force.^3^ In recent decades, it has been prevalent to measure the microchanges in the microstructural, morphological and mechanical properties of cells for the cancer diagnosis microscopically. Therefore, it is of great value to investigate the mechanical properties of lung cancerous cells, which potentially regard as a marker to detect lung cancer disease for clinical or medical diagnosis and treatment.^4^, ^5^, ^6^

The most widely developed methods for cell mechanics include atomic force microscopy (AFM), parallel‐plates rheometry, magnetic twisting cytometry (MTC), cell monolayer rheology (CMR), particle‐tracking microrheology (PTM), and the optical stretcher (OS).^7^ Among these approaches, AFM can detect morphological and mechanical properties of conductive and non‐conductive materials in liquid or air almost non‐destructively.^8^, ^9^ In the case of biological materials, especially for cells, AFM provides direct observation to maintain the physiological state of live cells in real time on the atomic scale with high force sensitively and precise spatial resolution.^10^, ^11^, ^12^ In addition, the AFM‐based indentation method has the unique advantages of small deformation and less damage to live cells. Recently, Dufrene outlined the applications, limitations and future opportunities of biological systems for diverse range of AFM imaging modes, including the contact mode, dynamic mode (tapping or oscillation mode), FD curve‐based imaging mode, molecular recognition imaging mode, multifrequency imaging mode, high‐speed imaging mode and correlative imaging.^13^

However, there are a variety of factors to affect the result acquisition of mechanical properties of cells, which mainly include biological variability and technical inaccuracy.^14^ The technical causes include instrumental errors, distinctive data acquisition and various data analysis. Instrumental errors mainly due to the inaccuracies in the deflection sensitivity, spring constant, tip geometry and size.^7^, ^14^, ^17^ Data acquisition influences owing to the various experiment conditions, for instance, sample position (e.g. cell central body or periphery),^7^ substrate stiffness,^25^, ^26^ loading force,^7^, ^15^, ^16^, ^21^, ^22^ loading rate,^7^, ^15^, ^17^, ^21^, ^22^ loading time,^21^, ^22^ relaxation time,^23^ biochemical and physical cues in surroundings^24^ like temperature,^7^ drugs^16^, ^19^, ^24^ etc. Data analysis is partly influenced by the applied theoretical models.^7^, ^14^, ^23^, ^32^, ^33^ Wu et al. not only compared the various measurement methods of cell mechanics, but also discussed reasons for the variations of measurement results, which include the stress, rate, probe's geometry, measurement location and the extracellular microenvironment.^7^ Guz et al. performed the effects of cone and radius sphere probes, Hertz/Sneddon models and Brush models on elastic modulus for human cervical epithelial normal and cancerous cells.^15^ Weber et al. determined the mechanical response of endothelial cells by employing loading rates of 0.5−20 μm/s and loading force of 100−10,000 pN with a sharp, four‐sided pyramidal indenter and indicated that the increase of stiffness with higher loading rates and higher loading force.^19^ Martens et al. investigated the effects of the blebbistatin and Y‐27632 drugs on fibroblast cells for 30–60 min using AFM and concluded that the blebbistatin changed the morphological and mechanical properties whereas Y‐27532 did not present any visible effects.^20^ Mathur et al. identified that the elastic properties of three various cell types vary significantly (*E*_endothelial cells_ < *E*_cardiac muscle cells_ < *E*_skeletal muscle cells_) and the relationship between the elastic response to the probe geometry had also been explored.^16^ McPhee et al. found that the statistical differences in elasticity of 3T3fibroblasts in three surroundings (Fn‐coated, PLL coated and uncoated glass) with various culture time of 1 day and 3 days.^24^ Melzak et al. discussed the relaxation times of biomolecules and cell components in relation to QCM and AFM techniques.^23^ Efremov et al. processed that the adhesion force and Young's moduli of Vero cells increased with the increase of indentation rate, contact time and indentation depth.^21^ Nawaz et al. developed an optical trap to found that the elastic moduli increased with the loading rate according to a weak power law at larger indentation (>0.2 μm).^22^ Three f‐actin‐disrupting drugs of Cytochalasins B, D and Latrunculin A played an important role on the mechanical properties in 3T3 and NRK fibroblasts by using the fluorescence microscopy and atomic force microscopy.^26^ The substrate rigidity (stiff and soft substrates) has been discovered to regulate the mechanical properties of cells, such as the adhesion force and migratory ability.^25^, ^26^ A range of recent developed mechanical models to characterise the mechanical properties of living cells has been examined.^7^, ^14^, ^23^, ^32^, ^33^ Sanchez et al. compared the three linear elastic, linear viscoelastic and power‐law rheology (PLR) models of HeLa and NIH 3T3 cells based on experimental force–distance curves (FDC) and concluded that the 3D PLR model might be more suitable to be applied in mammalian cells with relatively large deformations above 1 μm.^29^ Zhou et al. focused on the combination of the interpretation of AFM‐based FDC mechanical nano‐indentation measurement by different models and the finite element method (FEM) simulations.^30^ Therefore, it has been more and more essential to explore various influential factors to affect the result acquisition of mechanical properties of cells, especially during the data acquisition process.

In this paper, we mainly evaluated the measurement parameters during data acquisition process, which include indentation forces (nN), indentation speed (μm/s), dwell time (s), retraction time (s), Z length (μm) and solutions. The effects of measurement parameters on the nuclear central Young's moduli of a single live A549 cell were investigated in vitro using the AFM‐based force spectroscopy mode. In order to measure the characteristics of cancerous cells more comprehensively and precisely, the nano‐indentation experiments were carried out on different ranges of multiple effective parameters to select appropriate measurement values for future research. Moreover, the morphological characteristics were examined by combining with inverted optical microscopy, immunofluorescence, scanning electron microscopy and atomic force microscopy. Therefore, this research may have the potential significance to provide reference for the standardised detection of single cancerous cell in vitro based on AFM methodologies.

## 2 EXPERIMENTAL DETAILS

### 2.1 Cell culture and sample preparation

Lung cancerous cells (A549, the Cell Bank of Type Culture Collection of Chinese Academy of Science, China) were incubated in Dulbecco's Modified Eagle Medium (DMEM medium, Gibco Company, USA) with 10% foetal bovine serum (FBS, Hyclone Laboratories Inc., USA) and 1% penicillin‐streptomycin (Thermo Fisher Scientific, USA). Before the AFM experiment, 1 mL trypsin (Gibco Company, USA) was utilised to disperse cells and the diluted cells were centrifuged with 1000 rpm for 5 min and cultivated on cover glasses with a thickness of 0.13−0.17 mm in a 38 mm culture dish at 1 × 10^4^ cells densities in an incubator (IP610, Yamato, Japan) at the temperature of 37℃ with 5% CO_2_ for 24 h. Phosphate buffer saline (PBS, Gibco Company, USA) was utilised to clean experimental samples for triple times to remove the impurities and dead cells.

### 2.2 Inverted optical microscopy

The morphological properties of A549 cells can be evaluated using the inverted optical microscope (NIKON, Japan). A 20× objective was utilised for optical images at room temperature in super clean environment.

### 2.3 Immunofluorescence

A549 cells were diluted uniformly on cover glasses in 38 mm culture dish for 24 h, washed with PBS for three times, cured with 4% glutaraldehyde buffer for 10 min, treated with Alexa Fluor488^®^ phalloidin (Cell Signaling Technology, USA) for 30 min and subsequently stained with 4′,6‐diamidino‐2‐phenylindole (DAPI, Cell Signaling Technology, USA) for 30 s in dark at room temperature. Using the fluorescence mode of the inverted optical microscope (NIKON, Japan) with a 50× objective, cell membranes were shown as green colour while the cell nucleus appeared blue colour in fluorescent images.

### 2.4 Scanning electron microscopy

A scanning electron microscope (SEM, Quanta 250 FEI, USA) is used to demonstrate the morphological cytoskeleton properties in nano‐microscale for cured A549 cells. SEM analysis was implemented in high vacuum (HV) mode (4000× magnification, spot: 3.0, HV: 7.00 kV, chamber pressure: 1.62 × 10^−3^ Pa). Before SEM imaging, the cells were cultivated on the cover glasses for 24 h, rinsed with PBS, cured with 4% glutaraldehyde buffer at 4°C overnight, dehydrated with increased concentration of ethanol (50%, 70%, 80%, 85%, 90%, 95% and 100%) for 15 min, immersed in the tert‐butanol at −20°C for 12 h, and sputtered with a 5 nm gold layer.

### 2.5 Atomic force microscopy

The morphology characteristics and mechanical properties of live A549 cells were measured by the AFM (Nanowizard 3, JPK instruments, Germany). All AFM experiments were conducted at fixed room temperature of 23℃ in a clean room with normal CO_2_ levels. PBS was applied for the unified medium during AFM measurement in order to eliminate the effects of various culture mediums and serum concentrations. For each set of measurements, over 10 cells were measured for at least 5 times.

Generally, cantilever stiffness with values ranging from 0.01 to 0.3 N/m are utilised for cell mechanics. Thus, the MLCT‐A (Bruker, USA) was utilised for the AFM measurement with nominal spring constant of 0.07 N/m and has a triangular cantilever with four‐sided pyramidal tips. Before nano‐indentation experiments, the probe was required to be calibrated using the thermal noise method based on the AFM force spectroscopy mode. The practical deflection sensitivity and practical spring constant were calibrated as 17.82 nm/V and 0.072 N/m during the nano‐indentation measurement.

**FIGURE 1:**
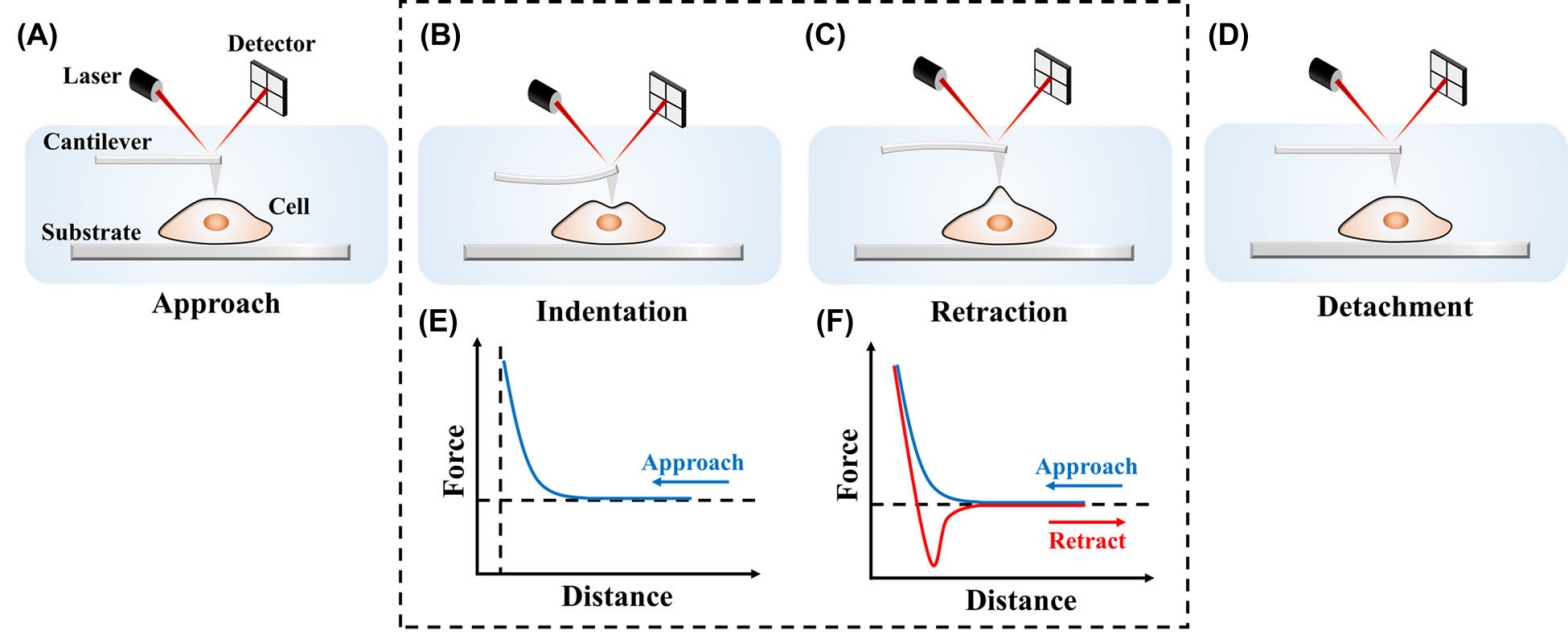
Schematic diagram of the vertical tip movement during the approach and retract parts of a single live cell by AFM: (A) diagram of approach; (B) diagram of indentation; (C) diagram of retraction; (D) diagram of detachment; (E) depiction of force–distance curve of the cantilever approaching to a single live cell by AFM pixel‐by‐pixel; (F) depiction of force–distance curves of the cantilever retracting to a single live cell by AFM pixel‐by‐pixel

The morphology properties were measured using a force–volume (FV) measurement^28^ of quantitative imaging mode (originally termed as QI mode) with 256 × 256 pixels while scanning live cells. The mechanical properties were characterised using force spectroscopy mode. Central nucleus region of live cells was measured during the nano‐indentation experiments in order to eliminate cellular anisotropy and substrate artefacts. The schematic diagram of the vertical tip movement of a single live cell using AFM primarily divided into four phases: approach phase, indentation phase, retraction phase and detachment phase (as shown in Figure 1A–D). When the AFM scanning, the morphology images of live A549 cells were obtained with the force–volume measurement. Figure 1E and F illuminated the force–distance curves of the cantilever approaching and retracting to a single live cell. The blue line expressed the approach curve while the red line described the retract curve.

### 2.6 Theoretical modelling

Mechanical properties and behaviour of cells offer an alternative tool to indicate the interior microstructure and characters inside cells. The mechanical properties of cells were characterised by AFM‐based force spectroscopy. Young's moduli were calculated from the experimental force–depth curves by fitting with the Sneddon model.^31^ The equation of Sneddon model is expressed as:

(1) $$F=\frac{{2E\delta }^{2}\tan\theta }{{\pi (1-v}^{2})}$$

where *F, δ, E, ν* and *θ* denote indentation force, indentation depth, Young's modulus, Poisson's ratio and half angle of the conical probe, respectively. Poisson's ratio is set to 0.5 for cells.^6^, ^28^ The measurement parameters include indentation forces (nN), indentation speed (μm/s), dwell time (s), retraction time (s) and Z length (μm). Z length implies the motion range in vertical direction and can be set to varied range in system. In the following experiment, the effects of the measurement parameters on the central Young's moduli were analysed by fitting the force–depth curves using the Sneddon model.

### 2.7 Data analysis

JPK SPM Data Processing software (SPM−6.0.65) was performed for extracted and processed the force–distance curves from AFM nano‐indentation experiments. The Sneddon model was implemented for the analysis of the approach curve. Origin Pro 9.0, Microso Visio 2010 and Excel 2007 were implemented for statistical analysis and data plotting. Matlab R2014a was applied to fit the force–distance curves.

## 3 RESULTS

### 3.1 Morphological properties of A549 cells

**FIGURE 2:**
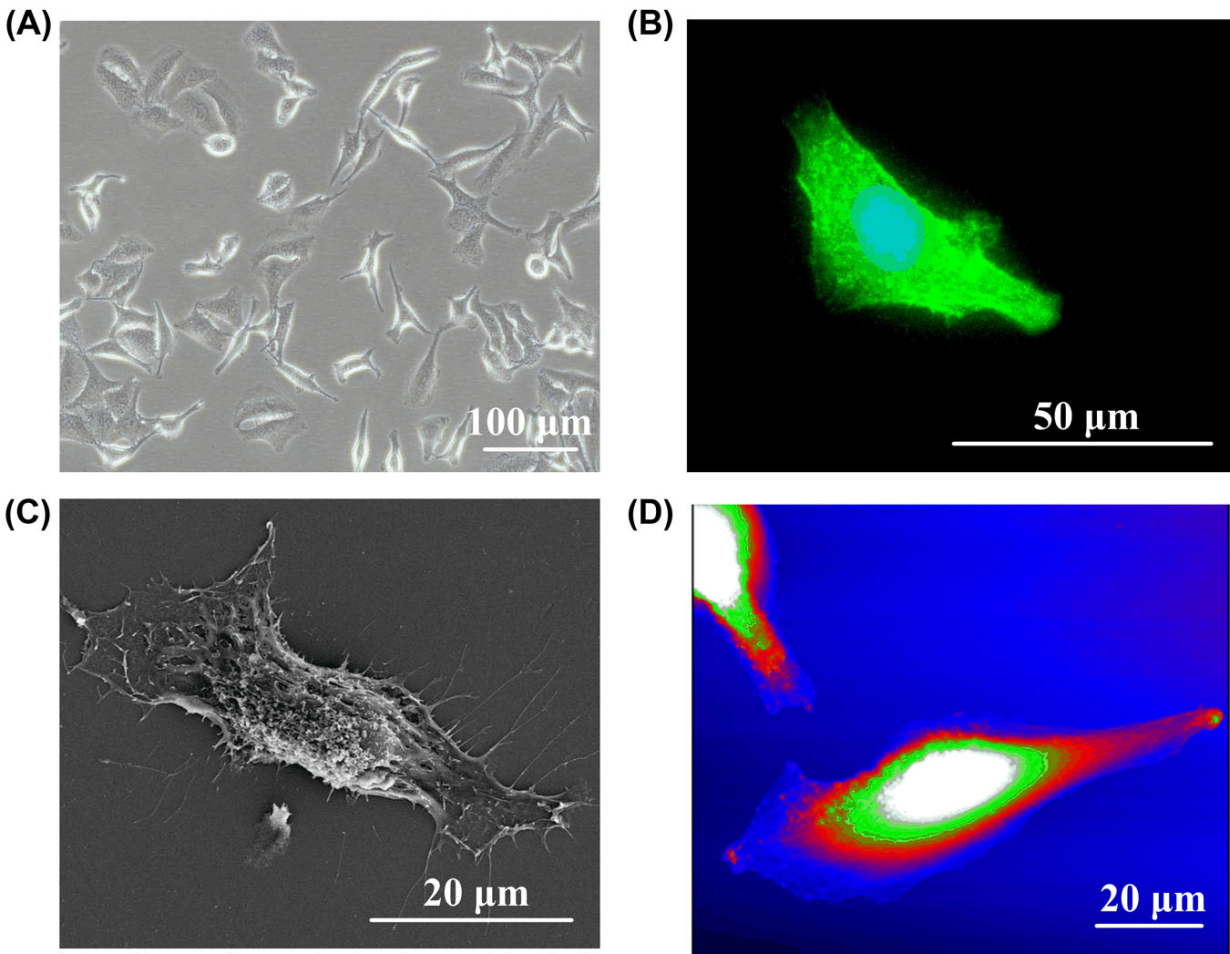
Morphological images of a single live A549 cell: (A) an inverted optical image with a 20× objective; (B) a fluorescent image with a 50× objective; (C) a SEM image with electron beam voltage of 7.00 kV and chamber pressure of 1.62 × 10^−3^ Pa in HV mode; (D) an AFM image with the setpoint of 1.0 nN

Morphology plays a significant role on the surface characteristics of live cells. It can be observed from Figure 2 that the morphological characteristics of cells were evaluated by inverted optical microscopy, immunofluorescence, scanning electron microscopy and atomic force microscopy, respectively. Combining AFM with inverted optical microscopy enables the observation of the morphological properties of live A549 cells, as shown in Figure 2A and D. The morphological properties of cured A549 cells were also measured using immunofluorescence and scanning electron microscopy, in order to provide more detailed information, as presented in Figure 2B and C.

**FIGURE 3:**
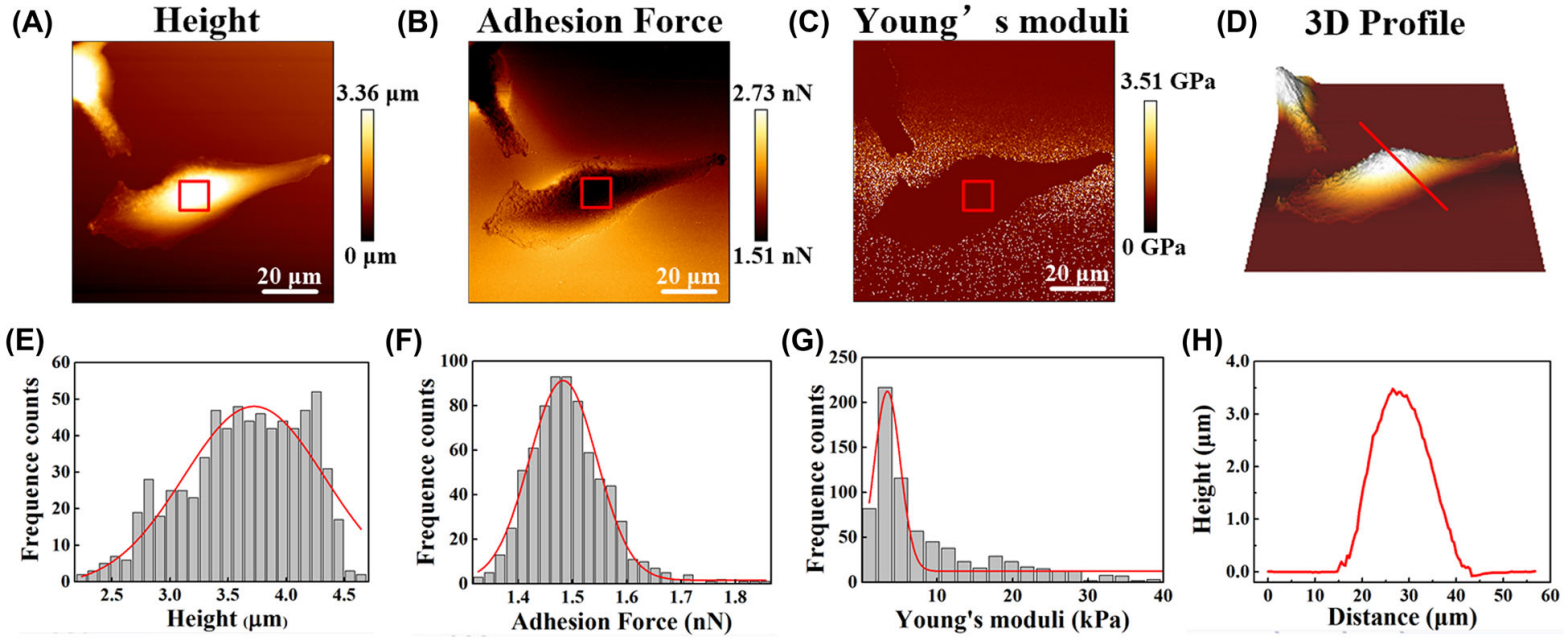
AFM force–volume measurement images of a single live A549 cell with 256 × 256 pixels: (A) height map; (B) adhesion force map; (C) Young's moduli map; (D) 3D profile map; (E) statistical distribution corresponding to the 10 μm × 10 μm selected statistical sections of height map; (F) statistical distribution corresponding to the 10 μm × 10 μm selected statistical sections of adhesion force map; (G) statistical distribution corresponding to the 10 μm × 10 μm selected statistical sections of Young's moduli map; (H) cross height profile of selected red cut line of 3D profile map. The red box in A–C is 10 μm × 10 μm. The red curve in E–G is the result after Gaussian Fitting. The red line in D is the cutting line for the cross section and the red curve in H is the corresponding curve to the cutting red line

As demonstrated in Figure 2, all morphological images of A549 cells exhibited slim spindle configurations with long length and narrow width. On the one hand, the morphology of the majority of live A549 cells were illuminated using inverted optical microscopy, as depicted in Figure 2A. The scale bar of the inverted optical image is 100 μm with a 20× objective. The general dimension ranges of A549 live cells vary from 50 to 90 μm in terms of the length scale while the widths change from 25 to 40 μm. On the other hand, the morphology of a single live A549 cell was scanned using AFM‐based force–volume measurement with a scale bar of 20 μm, as exhibited in Figure 2D. The shape of a single live A549 cell is configured as slim spindle and the dimension range is within the general dimension range of A549 cells. More details of AFM images such as height, adhesion force, Young's moduli and 3D profile were listed in Figure 3.

To be more precise, detailed microstructures of the cured cell, including cell internal cytoskeleton, nucleus distribution and microfilaments in the microvilli and pseudopodia, can be obtained from the fluorescent image and SEM images, as displayed in Figure 2B and C, respectively. The scale bar of the fluorescent image is 50 μm with a 50× objective. For SEM image, the scale bar is 20 μm with electron beam voltage of 7.00 kV and chamber pressure of 1.62 × 10^−3^ Pa. According to the fluorescent image, the nucleus of cured A549 cells manifested a blue oval shape in almost central area of cells after stimulating with 4’, 6‐diamidino‐2‐phenylindole (DAPI, Cell Signaling Technology, USA) for 30 s in dark at room temperature. In addition, the cell cytoskeleton was appeared as green colour in cell membranes’ area after treating with Alexa Fluor488^®^ phalloidin (Cell Signaling Technology, USA) for 30 min in dark at room temperature. Moreover, the microfilaments, microvilli and pseudopodia can be observed more clearly from the SEM images directly. Microfilaments and microvilli were disordered throughout the cell surface, which may cause the various roughness and complicated mechanical properties. It is feasible to observe the pseudopodia extensions with mass of clear microfilaments and microvilli from the SEM images, which has an important influence on the movement and migration of cells.

In order to follow the changes in surface topography closely and real time, AFM was selected to detect the morphology of live A549 cells and Bruker MLCT‐A probe was employed in this experiment. Height map and corresponding statistical distribution to the selected statistical sections of height map were obtained to estimate the height properties of single live A549 cell, as shown in Figure 3A and D. Similarly, adhesion map and corresponding statistical distribution to the selected statistical sections of adhesion force map were demonstrated in Figure 3B and E to examine the adhesion properties of single live A549 cell. The 3D profile with respective cross height profile were exhibited in Figure 3C and F. As depicted in Figure 3, a single A549 cell exhibited elongated spindle configurations with obvious pseudopodia extensions. The maximum height, maximum adhesion force and maximum roughness of the nuclear area of a single live A549 cell were 3.36 μm, 2.73 nN and 1.288 μm, respectively (shown in Figure 3A and B). In order to obtain specific dimension ranges of A549 cells, at least 10 A549 cells were measured with the same solutions and same batch. The length and width ranges of A549 cells were 50−90 and 25−40 μm, respectively.

### 3.2 Mechanical properties of live cells

#### 3.2.1 The effects of indentation force on the central Young's moduli

The indentation force is widely regarded as one of the most important measurement factors to cause the variations of the effective value of the Young's moduli of cancerous cells. The value of the maximum force applied might affect the effective value of the Young's moduli. Thus, the indentation force was set from 0.5 to 5.0 nN with the increment of 0.5 nN in this indentation measurement in order to explore the effect of the indentation force on the mechanical properties of A549 cells. The other measurement parameters of indentation speed, dwell time, retraction time and Z length were set to 2.0 μm/s, 0.0 s, 0.0 s and 5.0 μm, respectively. The indentation area is located at the central nucleus of A549 cells. The samples of cultured cells were put in the PBS solutions to eliminate the potential influence of solutions environment for the following experiment. In order to ensure the validity and accuracy, at least five force curves for over 10 cells were obtained in the indentation measurement.

**FIGURE 4:**
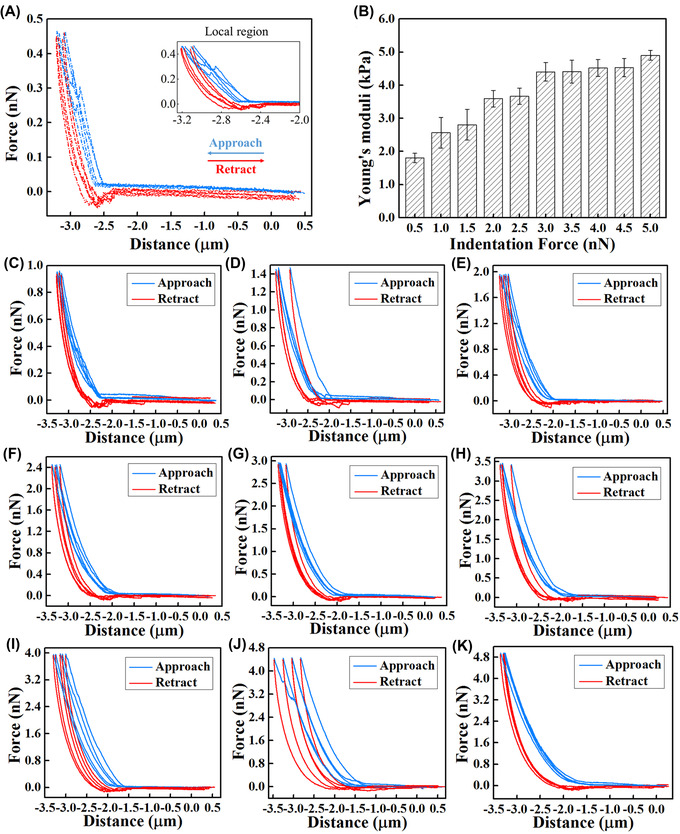
The effects of indentation force on the central Young's moduli. (A) Force–distance curves under 0.5 nN and the enlarged curves of the local region. (B) The effect of indentation force on the central Young's moduli. (C–K) Force–distance curves under 1.0, 1.5, 2.0, 2.5, 3.0, 3.5, 4.0, 4.5 and 5.0 nN, respectively

The results of the effects of indentation force on the central Young's moduli of A549 cells were summarised in Figure 4B, which derived from force–distance curves based on the JPK force Spectroscopy mode. The force–distance curves under 0.5 to 5.0 nN were demonstrated in Figure 4A, C–K, respectively. It can be observed that the average central Young's moduli of A549 cells increased with the increasing indentation force. With the increase indentation force from 0.5 to 5.0 nN, the average central Young's moduli vary from 1.80413 ± 0.14105 to 4.9045 ± 0.15013 kPa. This is due to the deformation of the interior cytoplasm, cytoskeleton, organelles and the nucleus in cells during nano‐indentation process of AFM probes. As the indentation force increased, the value of Young's moduli may increase as a result of squeezing the more rigid nucleus in the central region of cells. Therefore, the eventually average central Young's moduli of A549 cells were increased with the increasing indentation force.

#### 3.2.2 The effects of indentation speed on the central Young's moduli

The indentation speed is generally considered as the second significant measurement factor to influence mechanical properties of cancerous cells. In order to investigate the effect of the indentation speed on the mechanical properties of A549 cells, the indentation speed increased from 0.5 to 5.0 μm/s with the increment of 0.5 μm/s in this measurement. The other measurement parameters of indentation force, dwell time, retraction time and Z length were set to 1.0 nN, 0.0 s, 0.0 s and 5.0 μm, respectively. The indentation area is located at the central nucleus of A549 cells. The samples of cultured cells were put in the PBS solutions to eliminate the potential influence of solutions environment for the following experiment. In order to ensure the validity and accuracy, at least five force curves for over 10 cells were obtained in this indentation measurement.

**FIGURE 5:**
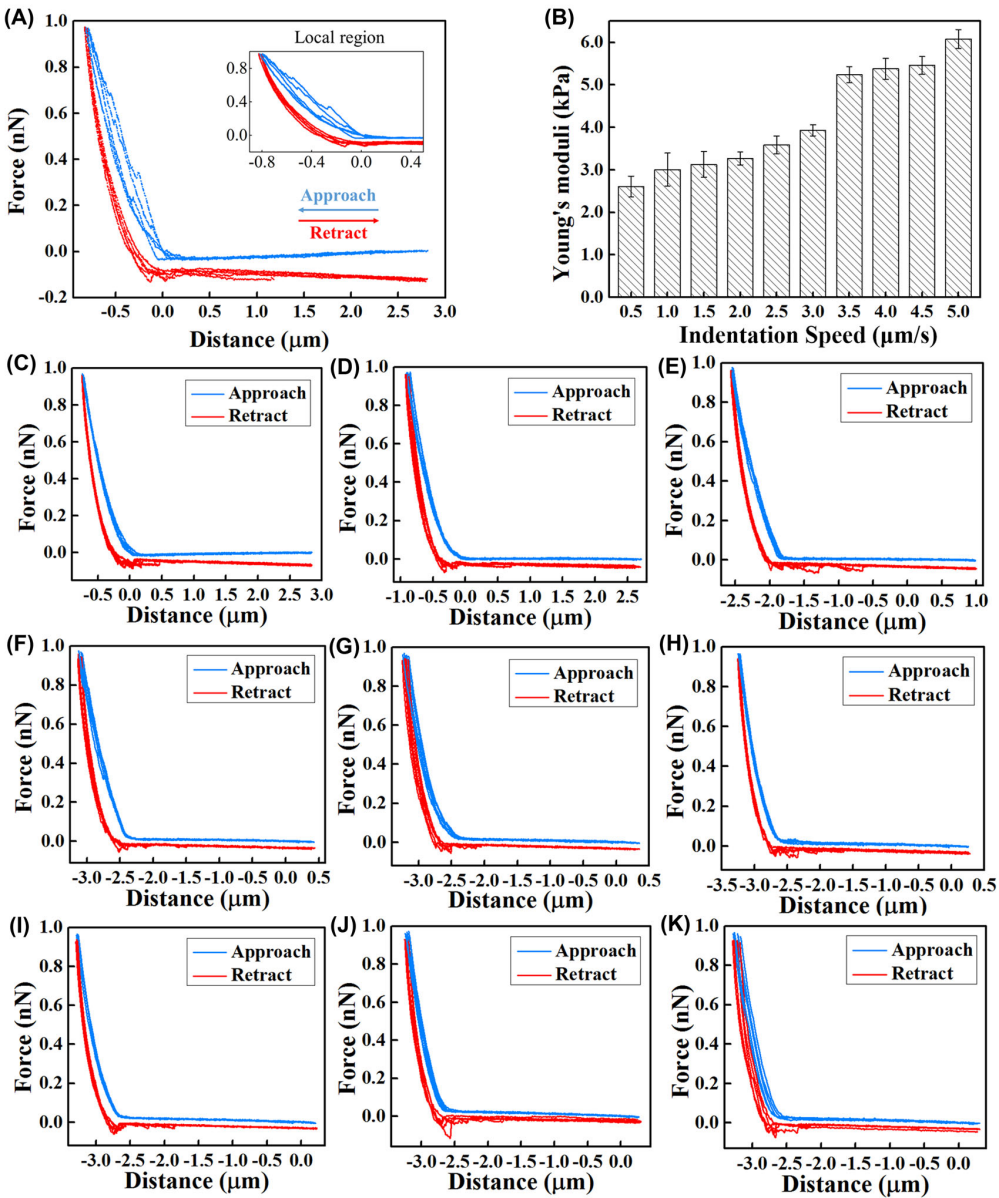
The effects of indentation speed on the central Young's moduli. (A) Force–distance curves under 0.5 μm/s and the enlarged curves of the local region. (B) The effect result of indentation speed on the central Young's moduli. (C–K) Force–distance curves under 1.0, 1.5, 2.0, 2.5, 3.0, 3.5, 4.0, 4.5 and 5.0 μm/s, respectively

After deriving from force–distance curves based on the JPK force spectroscopy mode, the results of the effects of indentation speed on the central Young's moduli were demonstrated in Figure 5. The force–distance curves with the indentation speed of 0.5−5.0 μm/s were illustrated in Figure 5A, C–K, respectively. From the Figure 5B, it can be seen that the Young's moduli of cells increased with the increasing indentation speed. With the indentation speed increased from 0.5 to 5.0 μm/s, the average central Young's moduli varied from 2.6 ± 0.245 to 6.082 ± 0.222 kPa. As the indentation speed increased, it is obvious to squeeze the cell more rapidly, as well as the interior cytoplasm, cytoskeleton, organelles and the nucleus in cells may deform more instantly. It may increase the possibility to compress the more rigid nucleus in the central region of cells. Therefore, the eventually average central Young's moduli of A549 cells were increased with the increasing indentation speed during nano‐indentation process utilising the AFM force spectroscopy.

#### 3.2.3 The effects of dwell time on the central Young's moduli

The dwell time is supposed to be the potential vital measurement factor among a variety of measurement parameters to alter the mechanical properties of cells. In order to investigate the effect of the dwell time on the mechanical properties of A549 cells, the dwell time increased from 0.5 to 5.0 s with the increment of 0.5 s in this measurement. The other measurement parameters of indentation force, indentation speed, retraction time and Z length were set to 1.0 nN, 2.0 μm/s, 0.0 s and 5.0 μm, respectively. The indentation area is located at the central nucleus of A549 cells. The samples of cultured cells were put in the PBS solutions to eliminate the potential influence of solutions environment for the following experiment. In order to ensure the validity and accuracy, at least five force curves for over 10 cells were obtained in this indentation measurement.

**FIGURE 6:**
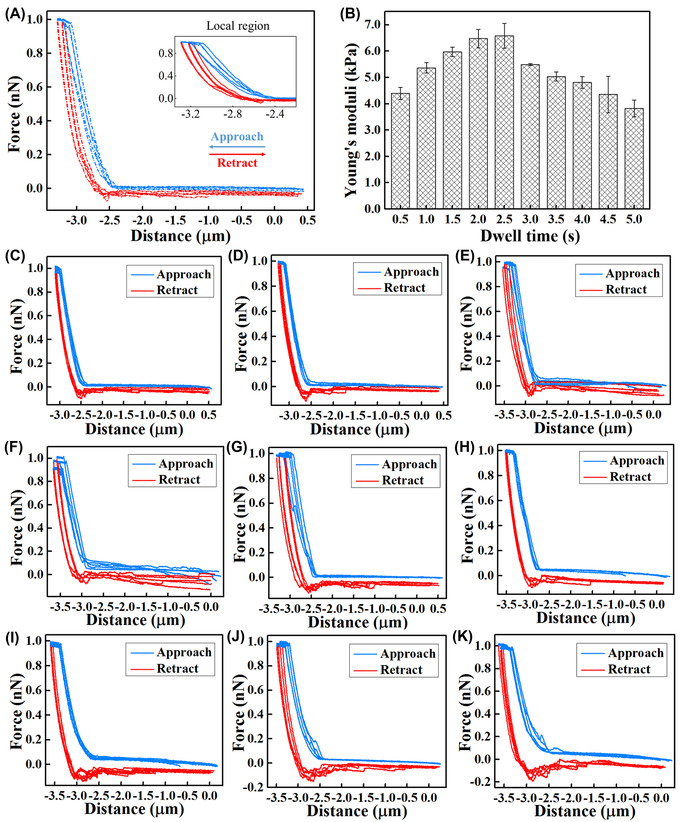
The effects of dwell time on the central Young's moduli. (A) Force–distance curves under 0.5 s and the enlarged curves of the local region. (B) The effect result of dwell time on the central Young's moduli. (C–K) Force–distance curves under 1.0, 1.5, 2.0, 2.5, 3.0, 3.5, 4.0, 4.5 and 5.0 s, respectively

After deriving from force–distance curves utilising the AFM‐based force spectroscopy mode, the results of the effects of dwell time on the central Young's moduli were presented in Figure 6. The force–distance curves with the dwell time of 0.5–5.0 s were demonstrated in Figure 6A, C–K, respectively. From the Figure 6B, it can be seen that the Young's moduli of cells increased first and then decreased with the increasing dwell time. The highest average central Young's moduli was 6.583 ± 0.466 kPa at the dwell time of 2.5 s. The reason for this phenomenon may due to the indentation phase with the pre‐set dwell time, which can be seen from the blue lines of Figure 6A, C–K. The blue lines of the indentation phase all have a short line parallel to the horizontal axis, which depend on the length of the dwell time. The dwell time may cause the complex interior deformation of cytoplasm, cytoskeleton, organelles and the nucleus in cells. With the increase the dwell time, the AFM probe may be more likely to squeeze the nucleus of cells. Beyond a certain dwell time (after 2.5 s in this measurement as shown in Figure 6B), the cell cytoplasm is more likely to rupture, which may cause the values of young's moduli decreased. Therefore, the eventually average central Young's moduli of A549 cells increased first and then decreased with the increasing dwell time during nano‐indentation process utilising the AFM force spectroscopy.

#### 3.2.4 The effects of retraction time on the central Young's moduli

By comparing with retraction time, the dwell time is deemed to be the potential influential measurement factor to affect the mechanical properties of cells. In order to investigate the effects of the retraction time on the mechanical properties of A549 cells, the retraction time increased from 0.5 to 5.0 s with the increment of 0.5 s. The other measurement parameters of indentation force, indentation speed, dwell time and Z length were set to 1.0 nN, 2.0 μm/s, 0.0 s and 5.0 μm, respectively. The indentation area is the central nucleus of A549 cells. The samples of cultured cells were put in the PBS solutions to eliminate the potential influence of solutions environment for the following experiment. In order to ensure the validity and accuracy, at least five force curves for over 10 cells were obtained in this indentation measurement.

**FIGURE 7:**
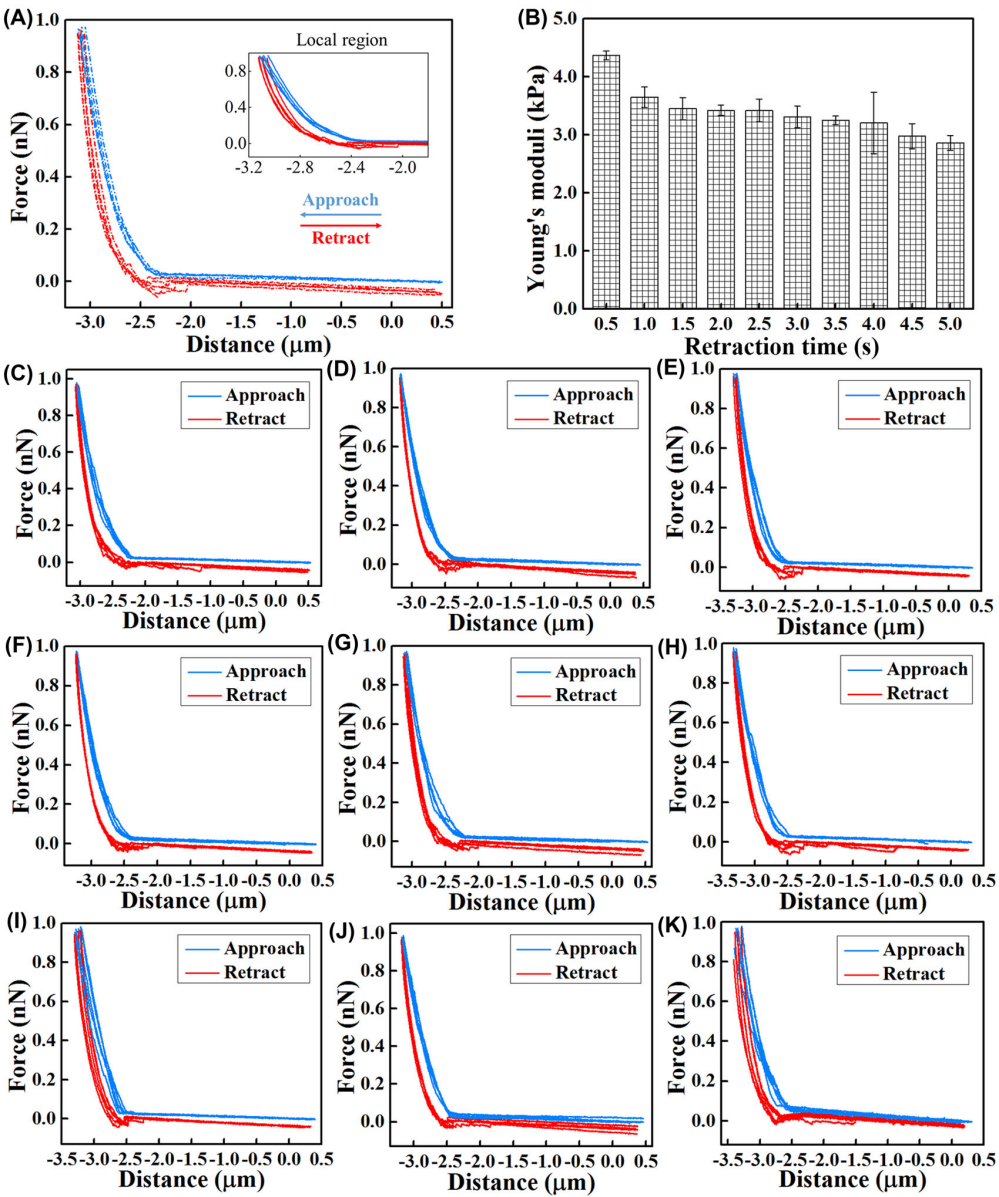
The effects of retraction time on the central Young's moduli. (A) Force–distance curves under 0.5 s and the enlarged curves of the local region. (B) The effect result of retraction time on the central Young's moduli. (C–K) Force–distance curves under 1.0, 1.5, 2.0, 2.5, 3.0, 3.5, 4.0, 4.5 and 5.0 s, respectively

Utilising the AFM‐based force spectroscopy mode, the results of the effects of retraction time on the central Young's moduli were demonstrated in Figure 7. The force–distance curves with the retraction time of 0.5–5.0 s were demonstrated in Figure 7A, C–K, respectively. From the Figure 7B, it can be seen that the Young's moduli of cells decreased with the increasing retraction time. With the retraction time increased from 0.5 to 5.0 s, the average central Young's moduli changed from 4.37 ± 0.08 to 2.86 ± 0.127 kPa. This phenomenon of the retraction time parameter is distinctive to results of the dwell time measurement. The complicated interior cytoplasm, cytoskeleton, organelles and the nucleus in cells may have been already deformed during the uniform indentation phase. So within the retraction phase, the increase retraction time may influence the adhesion force more significantly instead of the values of Young's moduli. With the increase the retraction time, the AFM probe expected to spend more time to departure the surface of cells. It may increase the possibility of the rupture of the cell structure, which may decrease the values of young's moduli. Therefore, the eventually average central Young's moduli of A549 cells decreased with the increasing retraction time during nano‐indentation process utilising the AFM force spectroscopy.

#### 3.2.5 The effects of Z length on the central Young's moduli

The measurement parameter of Z length is found out to be another potential impact factor to affect the mechanical properties of cells. Z length implies the motion range in vertical direction and can be set to varied range in AFM system. In order to investigate the effect of the Z length on the mechanical properties of A549 cells, the Z length increased from 4.0 to 10.0 μm with the increment of 1.0 μm. It is unable to obtain integrated data below the Z length of 4.0 μm. The other measurement parameters of indentation force, indentation speed, dwell time and retraction time were set to 1.0 nN, 2.0 μm/s, 0.0 s and 0.0 s, respectively. The indentation area is the central nucleus of A549 cells. The samples of cultured cells were put in the PBS solutions to eliminate the potential influence of solutions environment for the following experiment. In order to ensure the validity and accuracy, at least five force curves for over 10 cells were obtained in this indentation measurement.

**FIGURE 8:**
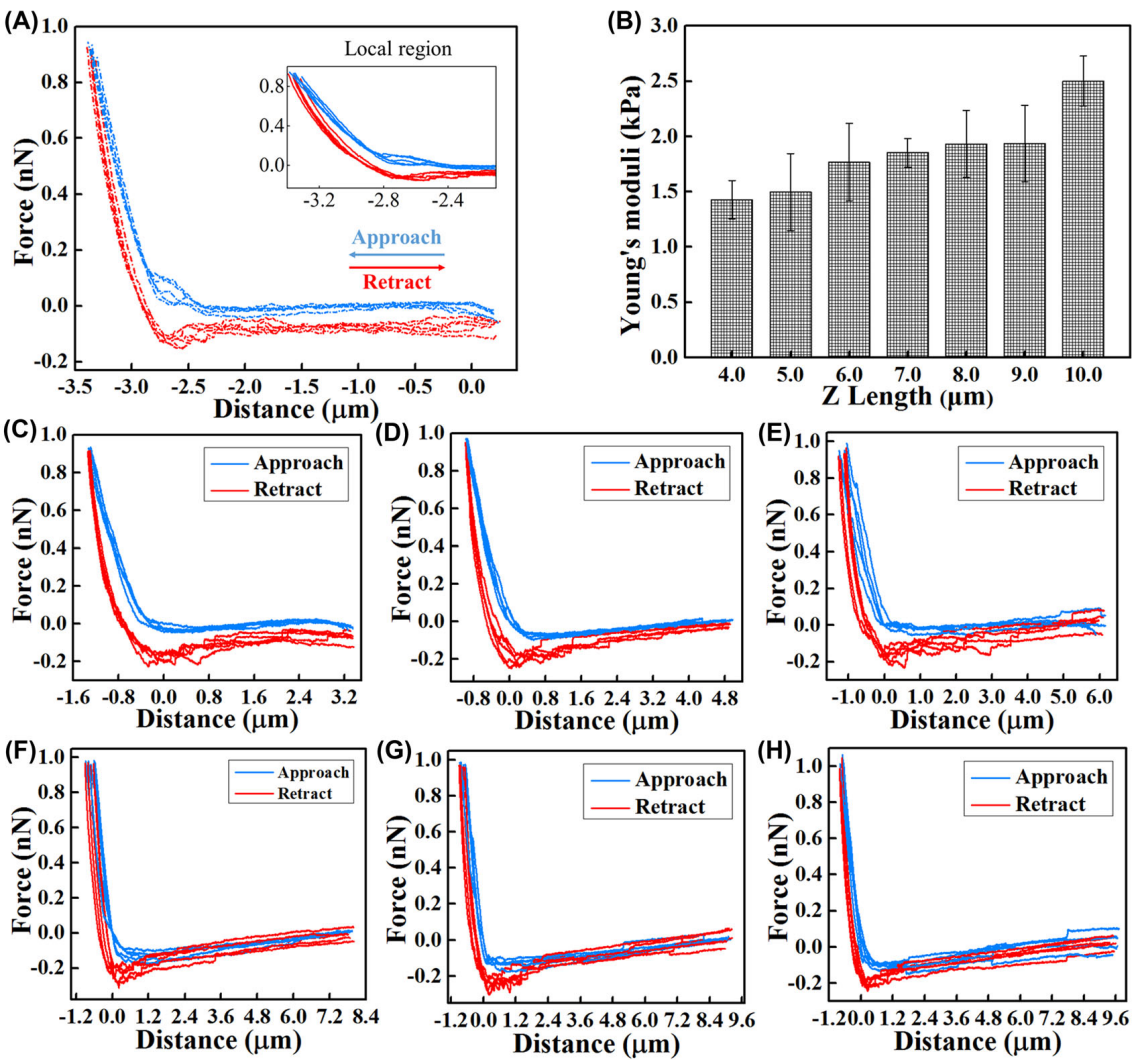
The effects of Z length on the central Young's moduli. (A) Force–distance curves under 4.0 μm and the enlarged curves of the local region. (B) The effect result of Z length on the central Young's moduli. (C–K) Force–distance curves under 5.0, 6.0, 7.0, 8.0, 9.0 and 10.0 μm, respectively

The results of the effects of Z length on the central Young's moduli were demonstrated in Figure 8. The force–distance curves with the Z length of 4.0–10.0 s were shown in Figure 8A, C–K, respectively. From the Figure 8B, it can be seen that the central Young's moduli of cells increased with the increasing Z length. With the Z length increased from 4.0 to 10.0 μm, the average central Young's moduli increased from 1.429 ± 0.173 to 2.502 ± 0.226 kPa. Z length implies the motion range in vertical direction and can be set to varied range in system, which generally can be a marker of length of the distance (μm) of horizontal axis. When the Z length is inferior to 4 μm, the force–distance curves is impossible to be presented entirely and the values of Young's moduli cannot be derived from the patchy force–distance curves. In this measurement, the variations of central Young's moduli of cells with the increasing Z length may be influenced by the anisotropy of cells.

#### 3.2.6 The effects of measurement solutions on the central Young's moduli

The measurement parameter of measurement solutions is proposed to be potential factor to influence the mechanical properties of cells. In order to investigate the effect of the measurement solutions on the mechanical properties of A549 cells, the samples of cultured cells were placed in the measurement solutions to implement the following experiment. The nutrient solutions of DMEM medium with different percentages of serum were set to 0% FBS, 10% FBS, 30% FBS, 50% FBS, 70% FBS, 90% FBS and 100% FBS, respectively. In addition, the PBS was applied to be compared with the nutrient solution without serum in order to characterise the effect of the solution to the mechanical properties of cells. The other measurement parameters of indentation force, indentation speed, dwell time, retraction time and Z length were set to 1.0 nN, 2 μm/s, 0.0 s, 0.0 s and 5.0 μm, respectively. The indentation area is located at the central nucleus of A549 cells. In order to ensure the validity and accuracy, at least five force curves for over 10 cells were obtained in this indentation measurement.

**FIGURE 9:**
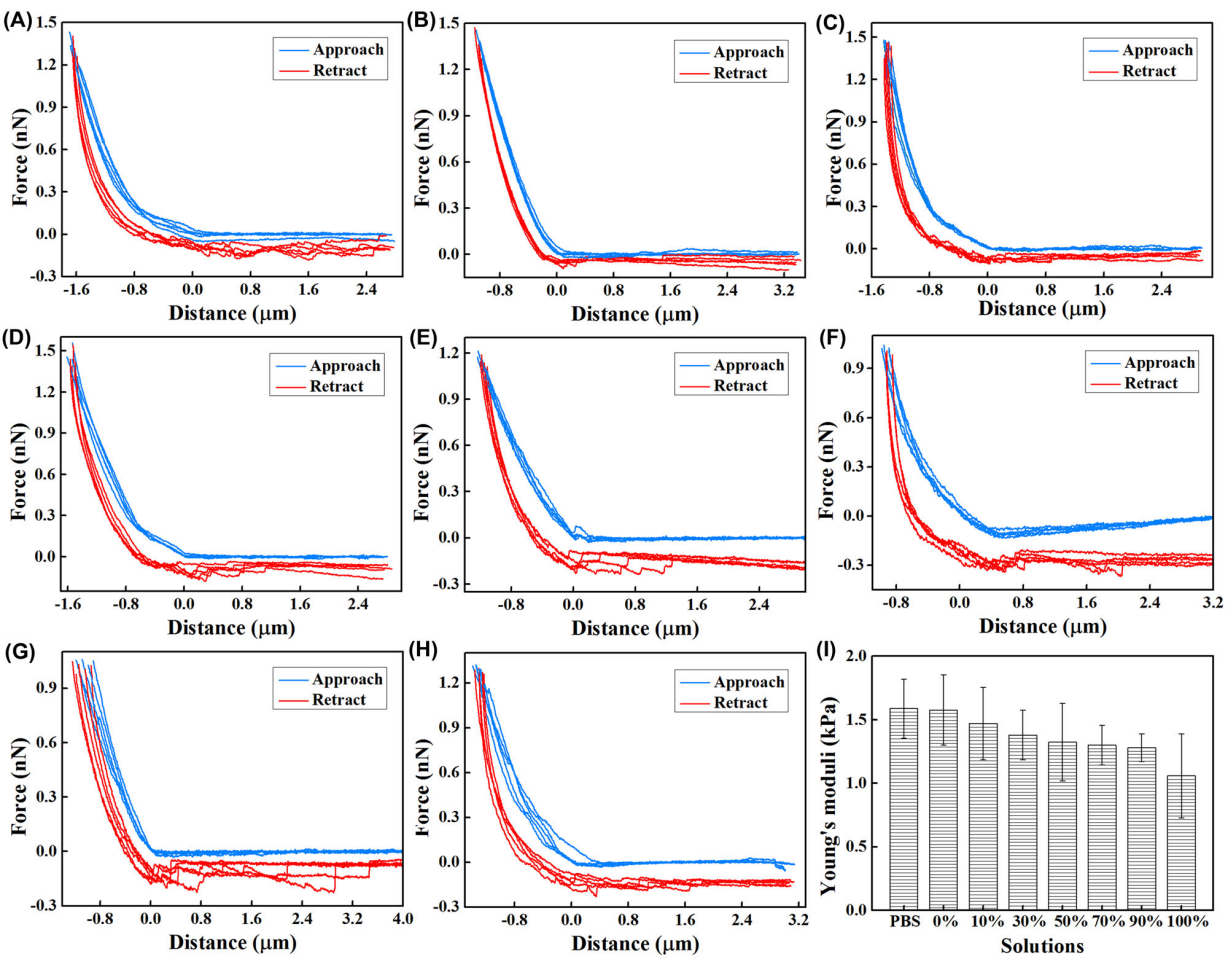
The effects of measurement solutions on the central Young's moduli: (A–H) force–distance curves under PBS, 0% FBS, 10% FBS, 30% FBS, 50% FBS, 70% FBS, 90% FBS, 100% FBS, respectively; (I) the effect result of nutrient solutions on the central Young's moduli

The results of the effects of solutions on the central Young's moduli were exhibited in Figure 9. The force–distance curves with the various measurement solutions were displayed in Figure 9A, C–K, respectively. From the Figure 9B, it can be seen that the Young's moduli of cells decreased with the increasing purity percentage of serum. While in PBS, the Young's moduli value was the highest of 1.588 ± 0.232 kPa. With the percentages of serum increased from 0% FBS to 100% FBS, the average central Young's moduli varied from 1.576 ± 0.277 to 1.059 ± 0.331 kPa. With the increase purity of serum, the solutions tend to be more viscous and then affects extend and retraction phase of AFM probe. In that case, the PBS solution was selected to be carried out in our previous measurement to reduce the influence of the various solutions in the nano‐indentation process.

## 4 DISCUSSIONS

We investigated the morphological properties and the influence of measurement parameters on the local elastic moduli of single live A549 cell in vitro based on the force spectroscopy using the atomic force microscopy. The measurement parameters in this nano‐indentation experiments include indentation forces (nN), indentation speed (μm/s), dwell time (s), retraction time (s), Z length (μm) and measurement solutions. Z length implies the motion range in vertical direction and can be set to varied range in system. The effects of the measurement parameters on the central Young's moduli were analysed by fitting the force–depth curves based on the Sneddon model. The results revealed that the Young's moduli of A549 cells increased with the higher indentation force, faster indentation speed, less retraction time, deeper Z length and lower purity percentage of serum. The Young's moduli of cells increased first and then decreased with the increasing dwell time.

However, there are many reasons for incorrect or inaccurate experimental results, such as non‐homogeneous character of cells, probe geometry and size, mechanical analysis model, substrate stiffness and different measurement parameters. It becomes prominent for substrate stiffness and complicated intracellular components when the indentation depths exceed 10% of the sample thickness.^33^ In order to reduce the influence of these factors, it is required to unify the above factors and pay more attention to the influence of measurement parameters on the mechanical properties. Therefore, Bruker MLCT‐A and uniform glass substrate were applied in this measurement. In addition, all the experiments focus on the nuclear central regions of live A549 cells because the Young's moduli were greatly influenced by the substrates at thinner cellular membranes.^34^ The experimental results obtained from the indentation force–distance curves were fitting by the Sneddon model. Although assumptions of homogeneity cannot be completely satisfied, the Sneddon model offers a good estimate of the Young's modulus for comparisons of cellular mechanics.^35^ Furthermore, at least five force curves for over 10 cells were measured for each experimental group in order to ensure the data validity and accuracy. The effects of measurement parameters on the local nucleus central Young's moduli of A549 cells are closely connect to complicated interior cytoplasm, cytoskeleton, organelles and the nucleus in cells. Consequently, the experimental results may indicate the interior microstructure of the cytoskeleton of cancerous cells based on Sneddon model by using the force spectroscopy mode with atomic force microscopy.

In the future, it is essential to standardise experimental operations and methodologies in order to avoid the personal equation and system deviation, which may cause inaccurate or incorrect results. For instance, the experimental results of Young's moduli may exhibit huge differences from references even in the uniform samples and identical equipment. Especially for cells, non‐homogeneous intracellular components inside cells are the main complex factors to cause the huge variations of the experimental results of cellular mechanical properties. Afterwards, the limitation of the current techniques may become another impact factor to lead to the low efficiency and accuracy of the nano‐indentation measurement. It has been more and more important to promote the scanning speed of large scale imaging in order to reflect the best and real physiological conditions of live cells and measure more live cells in one time, which usually spend almost 15 min to compete a 256 pixel × 256 pixel image of 100 μm × 100 μm nowadays. For A549 cells, the current maximum imaging of 100 μm × 100 μm allows to scan only one A549 cell in one time, which means it requires to spend more time to collect sufficient data and correspondingly reduce the efficiency. Moreover, it may be a future functions to reflect the interior microstructures and characters inside cells directly rather than speculation to indicate the potential reasons and changes inside cells according to computational models from nano‐indentation measurement. Moreover, universal theoretical and computational models are demanded for evaluation of a variety of cells or monolayer materials. It is ideal to establish the theoretical and computational model close to the real live cell instead of simplification and lots of limitations of assumptions. It is also essential to require an automatic and reliable algorithms in order to detect and analysis experimental results in batch mode rapidly.

## 5 CONCLUSIONS

We investigated the morphological properties and the influence of measurement parameters on the local elastic moduli of a single live A549 cell in vitro based on the force spectroscopy by using atomic force microscopy. On the one hand, the morphological characteristics were examined by combining with inverted optical microscopy, immunofluorescence, scanning electron microscopy and atomic force microscopy. On the other hand, the effects of the multiple measurement parameters on the central Young's moduli were analysed by fitting the force–depth curves based on the Sneddon model by utilising the AFM force spectroscopy mode. The results revealed that the Young's moduli of A549 cells increased with the higher indentation force, faster indentation speed, less retraction time, deeper Z length and lower purity percentage of serum. The Young's moduli of cells increased first and then decreased with the increasing dwell time. Therefore, this research may have the potential significance to provide reference for the standardisation detection of single cancerous cell in vitro based on AFM methodologies.

## CONFLICT OF INTEREST

The authors declare no conflicts of interest.
